# Superficial and deep lymph node dissection for stage III cutaneous melanoma: clinical outcome and prognostic factors

**DOI:** 10.1186/1477-7819-11-36

**Published:** 2013-02-04

**Authors:** Nicola Mozzillo, Corrado Caracò, Ugo Marone, Gianluca Di Monta, Anna Crispo, Gerardo Botti, Maurizio Montella, Paolo Antonio Ascierto

**Affiliations:** 1 Department of Melanoma, Sarcoma and Skin Cancer, Via Mariano Semmola, Naples, 80131, Italy; 2 Division of Melanoma and Skin Cancer, Via Mariano Semmola, Naples, 80131, Italy; 3 Division of Oncology, Via Mariano Semmola, Naples, 80131, Italy; 4 Division of Epidemiology, Via Mariano Semmola, Naples, 80131, Italy; 5 Division of Pathology, Via Mariano Semmola, Naples, 80131, Italy

**Keywords:** Cutaneous melanoma, Sentinel biopsy, Lymph node metastases

## Abstract

**Background:**

The aims of this retrospective analysis were to evaluate the effect of combined superficial and deep groin dissection on disease-free and melanoma-specific survival, and to identify the most important factors for predicting the involvement of deep nodes according to clinically or microscopically detected nodal metastases.

**Methods:**

Between January 1996 and December 2005, 133 consecutive patients with groin lymph node metastases underwent superficial and deep dissection at the National Cancer Institute, Naples. Lymph node involvement was clinically evident in 84 patients and detected by sentinel node biopsy in 49 cases.

**Results:**

The 5-year disease-free survival was significantly better for patients with superficial lymph node metastases than for patients with involvement of both superficial and deep lymph nodes (34.9% vs. 19.0%; *P* = 0.001). The 5-year melanoma-specific survival was also significantly better for patients with superficial node metastases only (55.6% vs. 33.3%; *P* = 0.001).

**Conclusions:**

Metastasis in the deep nodes is the strongest predictor of both disease-free and melanoma-specific survival. Deep groin dissection should be considered for all patients with groin clinical nodal involvement, but might be spared in patients with a positive sentinel node. Prospective studies will clarify the issue further.

## Background

Lymph node metastasis is a powerful predictor of recurrence and death in patients with cutaneous melanoma. Metastasis to regional lymph nodes develops during the course of the disease in approximately 30% of patients with cutaneous melanoma [1,2]. Radical lymph node dissection, defined as the removal of all lymph node levels of the involved basin, is recognized as the treatment of choice after histological or cytological evidence of lymph node involvement. For metastasis to inguinal lymph nodes, disagreement exists about the extent of surgical dissection and whether an iliac-pelvic lymphadenectomy is always mandatory. Many support the idea that iliac-pelvic metastatic involvement indicates systemic disease and that an aggressive surgical approach will not improve melanoma-specific survival [3,4]. Data on the long-term follow-up of patients with stage III melanoma (regional lymph node metastases) have demonstrated that about 20% of patients with involvement of deep (iliac and obturator) nodes were alive at 20 years, which suggests that surgical excision of nodal metastases is more than a staging or palliative procedure [5,6].

The aims of this retrospective analysis were to determine disease-free and melanoma-specific survival in the case of combined superficial and deep groin dissection, to identify the most important factors for predicting the involvement of deep nodes, and to describe differences in melanoma-specific survival and disease-free survival according to clinically or microscopically detected nodal metastases.

## Methods

Between January 1996 and December 2005, 520 patients underwent surgical lymph node dissection for metastases at the National Cancer Institute, Naples. Of these patients, 315 (60.6%) had metastases in lymph node basins other than the groin and 205 (39.4%) had inguinal lymph node metastases. Among the patients with inguinal lymph node metastases, combined superficial and deep groin dissection was considered the treatment of choice in patients with either a positive sentinel node or clinical groin disease. Superficial groin dissection only was limited to 72 patients for a variety of clinical reasons (such as anesthetic risk, age or life expectancy) and these cases were excluded from the present analysis.

The main clinical characteristics of the 133 patients included in the study are summarized in Table 1. The mean age was 50 years (range 21 to 83 years). The primary melanoma was located in the lower extremity in most patients (70.7%). The mean Breslow thickness of the primary melanoma was 4.0 mm and 77 melanomas (69.3%) were classified as T3 to T4. In 22 patients, histological findings of the primary melanoma were not available. In 84 patients (63.1%) (16 of whom had false negative sentinel nodes), adenopathy localized to the superficial groin area was clinically detected, with cytological confirmation of metastatic involvement, and in 49 patients (36.9%), micrometastatic disease was identified with sentinel node biopsy.

**Table 1:** Patient characteristics

**Factor**	**Characteristic**	**Number (%)**
Gender	Male	63 (47.4)
	Female	70 (52.6)
Age (years)	≤50	64 (48.1)
	>50	69 (51.9)
Primary site	Lower extremity	94 (70.7)
	Trunk	31 (23.3)
	Unknown	8 (6)
Clark level	III	35 (28.7)
	IV	64 (48.1)
	V	13 (9.8)
	Unknown	10 (8.2)
	NA	11
T stage	T1	9 (8.1)
	T2	25 (22.5)
	T3	36 (32.4)
	T4	41 (36.9)
	NA	22
Ulceration	Yes	57 (49.6)
	No	58 (50.4)
	NA	18
Method of diagnosis	Clinical disease	84 (63.1)
	Sentinel lymph node biopsy	49 (36.9)
Number of positive lymph nodes	1 (N1)	84 (64.1)
	2 or 3 (N2)	33 (25.2)
	≥4 (N3)	14 (10.5)
	NA	2
Extent of nodal involvement	Superficial	103 (77.4)
	Superficial and deep	30 (22.6)

NA, not available.

Disease was staged in all patients with the use of computed tomography (CT) and ultrasound to detect metastatic involvement of deep lymph nodes and distant metastasis. Patients with evidence of metastatic deep involvement were excluded from the present study.

### Surgical procedure

Surgery was performed according to a standard procedure in patients with either a positive sentinel node or clinical groin disease. The incision was carried from the apex of Scarpa’s triangle to about 5 cm superomedial to the superior anterior iliac spine. The dissection was carried through adipose tissue to the femoral vascular sheath, the femoral vessels were exposed, and the saphenous vein was sectioned at its femoral entrance and at the apex of Scarpa’s triangle. A separate incision was made in the lower abdominal musculature, leaving the inguinal ligament intact, to provide access to the retroperitoneal space. External iliac vessels were exposed by blunt dissection and retraction of the peritoneum. Dissection of the iliac lymphatic tissue was extended up to the common iliac bifurcation. The obturator dissection was brought through by gentle mobilization of the external iliac vein and the obturator nodes ventral from the obturator nerve were removed. The operation was completed with transposition of the sartorius muscle and insertion of a suction drain. Patients were invited to wear compression socks from the day after the operation up until six months later. No patient underwent adjuvant radiation or systemic therapy.

### Pathology

All lymph nodes were marked according to basin (femoral, iliac or obturator). The specimens were analyzed in a routine manner, with lymph nodes bisected or trisected according to their size, and stained with hematoxylin and eosin.

#### Follow-up and statistical methods

Patients were evaluated at follow-up visits every 3 months for the first 2 years, every 4 months during the third year and every 6 months thereafter. Follow-up consisted of clinical evaluation, ultrasound of the lymph node basin and liver, chest x-ray, and positron-emission tomography (PET)-CT scan, if deemed necessary.

Lymphedema was assessed by limb circumference measurements at standardized intervals, with limb volume measured preoperatively and during follow-up visits for all patients. The severity of lymphedema was assessed by comparing objective measures of volume discrepancy between the treated limb and the controlateral untreated limb. Estimation of the likelihood of local-regional relapse, and disease-free and melanoma-specific survival were calculated according to the Kaplan-Meier method for the entire patient population. Statistical differences between curves were calculated using the log-rank test.

Multiple regression analysis was performed with use of the Cox proportional hazard model, with a stepwise approach used to estimate the hazard ratio and relative 95% confidence intervals (95% CI) for each covariate. A *P* value of ≤0.05 was considered statistically significant. All time intervals were calculated from the date of stage III diagnosis. Statistical analysis was performed with SPSS (version 10.0; SPSS Inc., Chicago, IL, USA).

## Results

The patients were followed up for a median of 5.6 years.

### Nodal involvement

None of the 133 patients had clinical evidence of involvement of the deep lymph nodes at the time of initial staging with CT and ultrasound.

A total number of 2300 nodes were removed with a mean of 17.36 nodes removed for each patient (range 3 to 30). A mean of 11.82 nodes were removed from femoral dissection and a mean of 5.46 nodes from the ilio-obturator area.

Pathological evaluation of the 133 surgical specimens demonstrated that metastatic disease was limited to the superficial nodal basin in 103 patients (77.4%) and extended to the deep nodes in 30 patients (22.6%). Among 49 patients with tumor-positive sentinel nodes, only three (6.1%) had evidence of disease in the deep nodes, without involvement of other superficial nodes after completion of lymph node dissection. The primary melanoma was ulcerated in two of these three patients.

The remaining 27 cases (20.3%) with positive deep nodes, after lymph node dissection, belonged to the group of 84 patients with clinically positive inguinal nodes at diagnosis.

Postoperative morbidity was low. No patients died. Hemorrhage occurred in 2.3% of patients, wound infection and skin necrosis in 11.3%, and seroma grade 2 or 3 in 17.3% after suction drain removal.

Postoperatively, the rate of relapse in local lymph nodes was 9.7% (13 patients) and the rate of in-transit disease was 6.8% (nine patients). Long-term complications included paresthesias and chronic lymphedema. No patients referred paresthesias after 6 months. Lymphedema, which was usually mild, occurred in 40% of patients; periodic therapy with sequential compression devices was used in 9% of patients [7].

### Disease-free survival

At the time of the most recent follow-up, 84 patients had relapsed disease (local, in-transit or regional nodal metastases in 14, and loco-regional and distant metastasis in 73). The median time to relapse was 22.0 months (23.2 months for local-regional relapse and 21.5 months for distant metastasis). In 44 patients (75%), disease recurred within 24 months.

**Figure 1:**
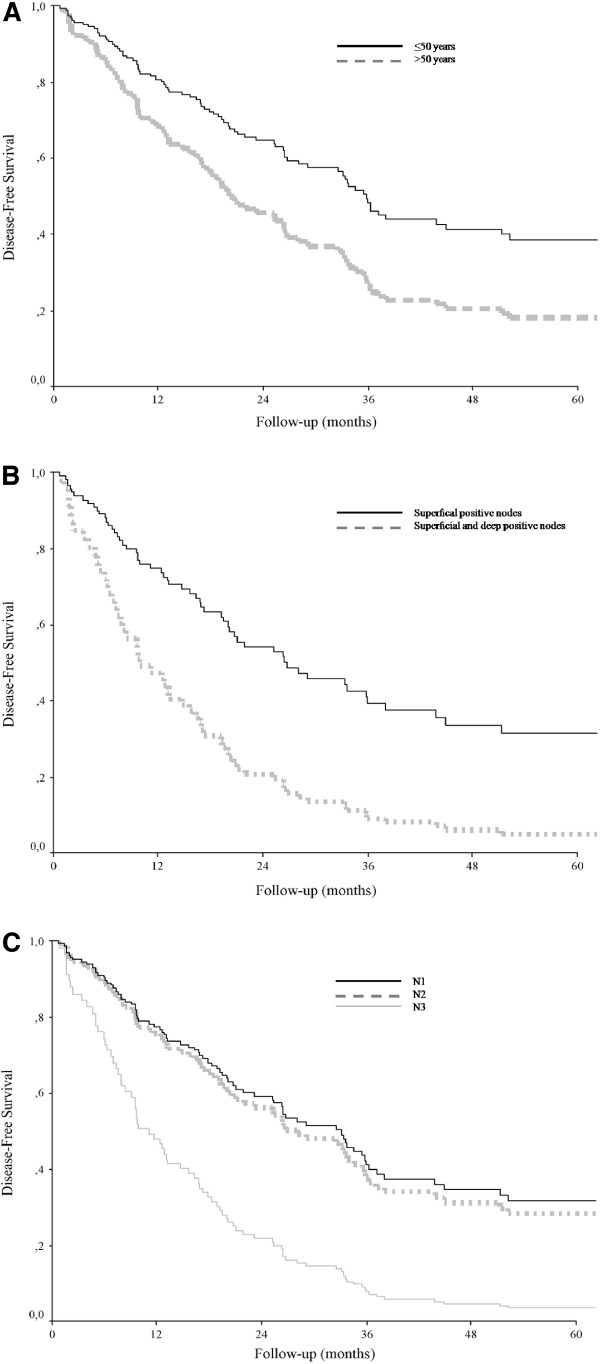
**Disease-free survival.** According to age (**A**), extent of lymph node involvement (**B**) and number of positive lymph nodes (**C**). *P* values determined with use of log-rank test.

Kaplan-Meier analysis of disease-free survival was undertaken according to clinical and pathological features (Table 2). The 5-year disease-free survival was significantly worse for patients older than 50 years than for patients 50 years or younger (28.9% vs. 45.3%; *P* = 0.008) (Table 2 and Figure 1A). An age of 50 years was selected as the cut-off point on the basis of previous studies of age as a prognostic factor [5].

**Table 2:** Kaplan-Meier analysis of 5-year disease-free and melanoma-specific survival

**Clinical and pathological factors**	**Disease-free survival**	**Melanoma-specific survival**
**Age (years)**		
≤50	45.3	64.1
>50	28.9	52.2
*P* value	0.0098	0.03
**Method of diagnosis**		
Sentinel lymph node biopsy	41.3	69.3
Clinical	29.2	43.7
*P* value	0.4	0.004
**Primary site**		
Trunk	22.6	45.2
Lower extremity	41.5	62.7
Unknown	37.5	50
*P* value	0.1	0.5
**T stage**		
T1 to T2	52.9	73.5
T3	27.8	47.2
T4	41.5	65.8
*P* value	0.2	0.4
**Ulceration**		
No	36.8	58.6
Yes	29.3	54.4
*P* value	0.6	0.2
**Number of positive lymph nodes**		
1 (N1)	41.7	65.5
2 or 3 (N2)	36.4	54.5
≥4 (N3)	7.1	14.3
*P* value	0.007	0.0009
**Extent of nodal involvement**		
Superficial	34.9	55.6
Superficial + deep	19.05	33.3
*P* value	0.001	0.001

The 5-year disease-free survival rate was significantly greater for patients who had positive nodes detected by sentinel node biopsy than for patients who had clinically detected adenopathy (41.3% vs. 29.2%; *P* <0.04).

The 5-year disease-free survival rate also differed significantly according to the extent of nodal involvement, with a rate of 34.9% for patients who had involvement of only superficial nodes, and 19.0% for patients who had involvement of superficial and deep nodes (*P* = 0.001) (Table 2 and Figure 1B). In addition, the number of metastatic lymph nodes was significantly associated with 5-year disease-free survival, with a rate of 41.7% for N1 patients (number of positive lymph nodes = 1) and 7.1% for N3 patients (number of positive lymph nodes ≥4) (*P* <0.007) (Table 2 and Figure 1C). Of 49 positive sentinel node cases, 93.5% had only one metastatic node and 84.8% of micrometastases were located in the subcapsular area.

Location of the primary melanoma, tumor classification (T stage) and ulceration were not significantly associated with 5-year disease-free survival.

### Melanoma-specific survival

**Figure 2:**
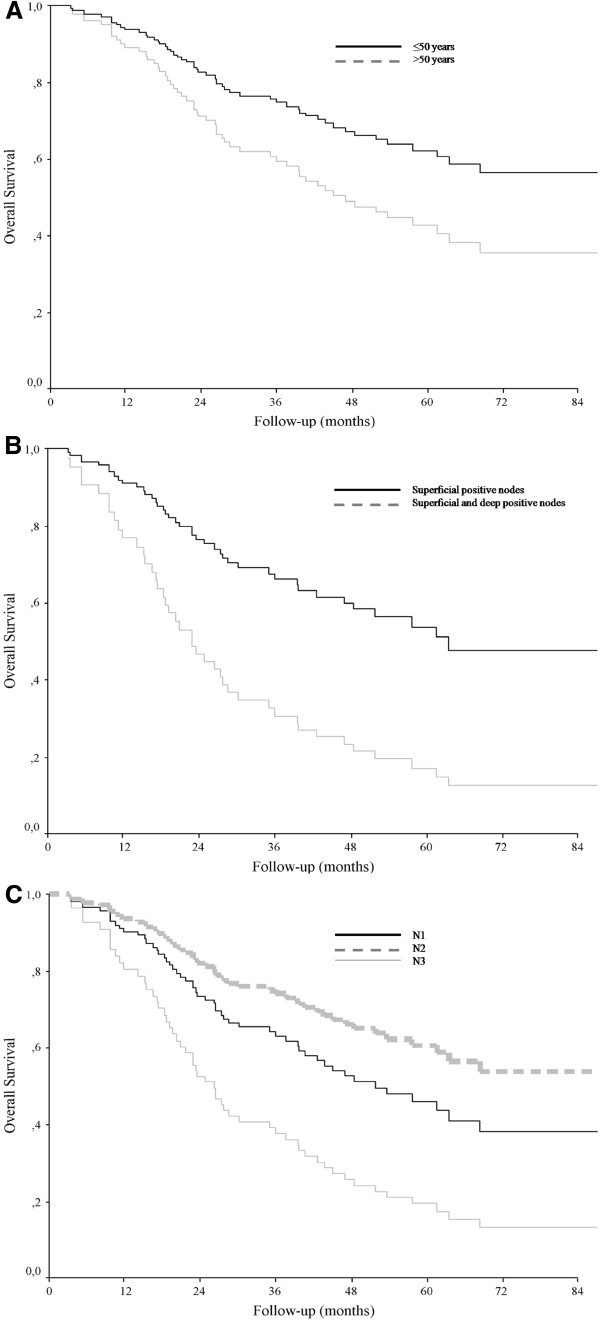
**Melanoma-specific survival.** According to age (**A**), extent of lymph node involvement (**B**) and number of positive lymph nodes (**C**). *P* values determined with use of log-rank test.

There was a significant difference in 5-year melanoma-specific survival according to age, with a rate of 64.1% for patients 50 years or younger and 52.2% for patients older than 50 years (*P* <0.03) (Table 2 and Figure 2A).

The difference in 5-year melanoma-specific survival between superficial and deep nodal involvement was significant (55.6% vs. 33.3%; *P* = 0.001) (Table 2 and Figure 2B). The number of positive lymph nodes also had prognostic importance, with a significant difference between 5-year survival for N1 and N3 disease (65.5% vs. 14.3%, respectively; *P* <0.0009) (Figure 2C). The 5-year melanoma-specific survival associated with N1, N2 and N3 classification was 70.8%, 54.5% and 14.3%, respectively.

Diagnosis of metastasis with the use of sentinel node biopsy was associated with better 5-year melanoma-specific survival than clinical diagnosis of metastasis (69.3% vs. 43.7%; *P* <0.004) (Table 2).

Location of the primary melanoma, tumor classification (T stage) and ulceration were not significantly associated with melanoma-specific survival (Table 2).

### Multivariate analysis

Multivariate analysis confirmed that involvement of deep nodes was associated with a significantly worse prognosis than involvement of superficial nodes only, with hazard ratios for 5-year disease-free survival and melanoma-specific survival of 2.6 (95% CI, 1.4 to 4.6; *P* = 0.001) and 2.8 (95% CI, 1.5 to 5.5; *P* = 0.002), respectively (Table 3). The number of involved lymph nodes also significantly correlated with prognosis, with a better outcome for N1 disease compared with N2 and N3 disease (Table 3).

**Table 3:** Multivariate analysis of 5-year disease-free survival and melanoma-specific survival

**Clinical and pathological factors**	**Disease-free survival**	***P *value**	**Melanoma-specific survival**	***P *value**
Method of diagnosis		0.6		0.01
Sentinel lymph node biopsy	1		1	
Clinical	1.1 (0.7 to 1.7)		1.7 (1.0 to 3.0)	
Primary site^a^		0.05		0.07
Trunk	1		1	
Lower extremity	0.5 (0.3 to 0.9)		0.6 (0.3 to 1.1)	
Unknown	1.1 (0.3 to 3.6)		21.7 (0.5 to 5.6)	
T stage		0.05		0.09
T1 to T2	1		1	
T3	2.2 (1.2 to 4.1)		2.5 (1.1 to 5.6)	
T4	1.6 (0.8 to 3.0)		1.6 (0.6 to 3.8)	
Ulceration^b^		0.3		0.06
No	1		1	
Yes	1.3 (0.7 to 2.3)		1.9 (1.0 to 3.7)	
Number of positive lymph nodes		0.006		0.003
1 (N1)	1		1	
2 or 3 (N2)	1.2 (0.6 to 2.2)		1.6 (0.7 to 3.5)	
≥4 (N3)	3.6 (1.6 to 7.8)		4.7 (1.9 to 11.5)	
Extent of nodal involvement		0.001		0.002
Superficial	1		1	
Superficial + deep	2.6 (1.4 to 4.6)		2.8 (1.5 to 5.5)	

Multivariate analysis also demonstrated significantly better 5-year disease-free survival for melanomas on a lower extremity than for those on the trunk (HR 0.5; 95% CI, 0.3 to 0.9; *P* = 0.05) and for T1 to T2 disease compared with T3 disease (HR 2.2; 95% CI, 1.2 to 4.1; *P* = 0.05). However, neither of these factors were significantly associated with melanoma-specific survival (Table 3).

The method of diagnosing lymph node metastasis (by sentinel node biopsy or clinical diagnosis) was significantly associated with melanoma-specific survival but not disease-free survival (Table 3). Ulceration of the primary melanoma was not significantly associated with either disease-free or melanoma-specific survival.

### Logistic regression analysis

Because of the strong association found between the extent of nodal involvement and melanoma-specific survival, we performed a logistic regression analysis with extent of nodal involvement as the dependent variable. We analyzed the odds ratios according to age, primary site, ulceration, method of diagnosis of metastasis and tumor classification (T stage). No significant associations were found.

## Discussion

In the pre-sentinel lymph node biopsy era, many studies showed that the extent or aggressiveness of regional surgical therapy did not have a significant impact on melanoma-specific survival, with the extent of nodal involvement at the time of diagnosis being the only predictor of outcome [8-10]. Sterne *et al.* have suggested that extensive lymph node dissection has a significant impact on both disease-free and melanoma-specific survival [11], and the findings of other studies have indicated that reducing the extent of surgery increases the risk of local failure and leads to lower survival rates [12-14].

Previous studies have also suggested that complete lymph node dissection not only improves local-regional control but also improves survival, particularly when the procedure is performed in a melanoma treatment center [15-19].

In our study, 33.3% of patients with involvement of deep nodes were alive 5 years after radical lymph node dissection and 19.0% were disease-free. Our data suggest that surgical dissection plays a major role in the staging of ilio-obturator nodal disease; prevents the morbidity of pelvic dissemination, which is associated with poor quality of life; and is related to 5-year survival for about one-third of patients [20-22].

One of the arguments against deep groin dissection is the associated morbidity. The rate of complications after superficial and deep groin dissection has been reported to be as high as 50% [23]. Infection, hemorrhage, skin-flap necrosis, wound dehiscence, paresthesia and lymphocele with chronic lymphedema have been the most commonly reported short and long-term complications [24-28]. Although both the risks and benefits of such dissection must be considered, many studies have shown a change in morbidity with modification to the extent of surgery. In a review of the literature, Hughes et al. found no evidence that deep groin dissection caused greater morbidity than superficial dissection [29]. Since 2001, the conservative surgical approach, without section of the inguinal ligament, has reduced postoperative morbidity, particularly postoperative pain.

Ten-year melanoma-specific survival rates range widely in the literature, reaching 40% in some reports, indicating the possibility of cure. Data from three major cancer institutions (Memorial Sloan-Kettering Cancer Center, York Avenue, NY, USA; Netherland Cancer Institute, Amsterdam, The Netherlands; and The University of Texas MD Anderson Cancer Center, Houston, TX, USA) have demonstrated that survival can be achieved in a substantial number of patients with deep nodal metastases, with 5-year survival rates ranging from 24% to 43% [9,14,23,30]. In these studies, the number of metastatic nodes, the thickness of the primary melanoma and ulceration of the primary melanoma were independent prognostic factors.

Sentinel node-positive disease in the groin was related to involvement of deep nodes in 6.1% of the patients in our study, a finding similar to that of Santinami et al., who reported that 8.6% of patients had involvement of nodes in the iliac basin [26]. Involvement of deep nodes cannot be predicted by histological or clinical features. Neither the degree of clinically detected nodal disease, as analyzed by Sterne *et al.*[11], nor the status of Cloquet’s node, as proposed by Essner [10], are reliable methods for predicting the presence of disease in pelvic nodes. Conventional imaging and PET have been reported to have limited utility [31,32]. In a retrospective review of the role of PET at the time of diagnosis of T2 to T4 melanoma, Clark *et al.* found that the imaging method was not useful in identifying early regional or distant metastases [32]. In the present series, patients with evidence of metastatic deep involvement revealed at pelvic CT scan were excluded. In these cases, surgical treatment has no staging purpose and the extension of dissection has no effect on overall survival [33].

Our analyses showed that involvement of deep nodes was the most important prognostic factor for patients with stage III melanoma. Multivariate analysis indicated that other prognostic factors for both disease-free and melanoma-specific survival were the number of positive lymph nodes, age and method of diagnosis (sentinel node biopsy or clinical).

The findings of the present study suggest that combined inguinal and pelvic lymph node dissection should be considered for patients with clinical evidence of groin nodal disease, as this approach achieved survival of 5 years for about one-third of patients in the presence of deep disease. Patients with a positive sentinel node might be spared ilio-obturator dissection owing to the low risk of nodal involvement in this area.

## Conclusions

The lymphatic spread of cutaneous melanoma represents a crucial aspect in outcome and worsens both disease-free and melanoma-specific survival. The presence of metastases in the deep nodes is the strongest predictor of both disease-free and melanoma-specific survival. Deep (iliac and obturator) groin dissection should be considered a therapeutic procedure for patients with clinical involvement of the inguinal lymph nodes, but might be spared in patients diagnosed with a positive sentinel node.

## Consent

Written informed consent was obtained from all patients to allow analysis of data for research purposes. A copy of the written consent is available for review as part of the Institutional Medical Chart by the Editor-in-Chief of this journal.

## Abbreviations

CI: Confidence intervals; CT: Computed tomography; PET: Positron-emission tomography.

## Competing interests

The authors declare that they have no competing interest. There is no external source of funding involved in the submitted article.

## Authors’ contributions

NM conceived the study and realized the technique. CC drafted the manuscript, helped to conceive the study and carried out the literature research. UM helped in the preparation of the manuscript. GDM carried out the literature review. AC performed statistical analyses. GB carried out the literature review and helped in the management of the patients. MM performed statistical analyses. PAA was involved in drafting the manuscript or revising it critically for important intellectual content. All authors read and approved the final manuscript.
